# Caudal lumbar spinal cysts in two French Bulldogs

**DOI:** 10.1186/s13028-018-0368-6

**Published:** 2018-03-01

**Authors:** Kiona Sharon de Nies, Ralph Alexander Edwards, Niklas Bergknut, Martijn Beukers, Björn Petrus Meij

**Affiliations:** 1 Department of Clinical Sciences of Companion Animals, Faculty of Veterinary Medicine, Utrecht University, Yalelaan 108, 3584 CM Utrecht, The Netherlands; 2 Department Neurology, North Downs Specialist Referrals, Bletchingley, UK

**Keywords:** Dorsal laminectomy, French Bulldog, Lumbar spine, Magnetic resonance imaging, Spinal cysts

## Abstract

**Background:**

Spinal cysts are rare findings in veterinary medicine, but they are increasingly recognized due to the availability of advanced imaging techniques. Extradural meningeal cysts in French Bulldogs have not been reported previously and arachnoid cysts (diverticula) have not been reported at the caudal lumbar (L6–L7) region in dogs.

**Case presentation:**

Two French Bulldogs, aged 5 and 8 years, were referred for evaluation of lower back pain and bilateral hind limb neurological deficits. Neurologic examination revealed ataxia and postural deficits in both dogs. Magnetic resonance imaging (MRI) showed cauda equina compression due to a cyst-like lesion at the level of L6–L7 in both cases. The dogs underwent dorsal laminectomy and the meningeal cyst was completely removed in one dog and in the other dog the spinal arachnoid diverticula was marsupialized. In Case 1, histopathology of the cysts was performed and MRI was repeated. Both dogs were pain free during follow-up evaluations.

**Conclusions:**

Based on radiological, intra-operative and histopathological findings, the first case was diagnosed as a meningocele connected by a pedicle to the caudal tip of the dural sac forming a dural diverticulum categorized as an extradural spinal cyst type Ib, and Case 2 as a type III intradural arachnoid diverticula. It is concluded that spinal cysts should be included in the differential diagnosis of cauda equina syndrome and lower back pain in French Bulldogs. Results of these cases may be useful for diagnostic and treatment management.

## Background

A cyst is defined as ‘a closed epithelium-lined sac or capsule containing liquid, air or a semi-solid substance’ [1]. Cystic-like lesions that lack an epithelial lining are not true cysts and therefore the term diverticula is a more appropriate term [1, 2]. Spinal cysts and diverticula associated with spinal cord dysfunction are rare conditions in veterinary medicine, but they are increasingly recognized due to the availability of advanced imaging techniques like magnetic resonance imaging (MRI) and computed tomography (CT) [3, 4]. The aetiology of spinal cysts remains unclear but most are considered congenital lesions with a genetic predisposition, although acquired/traumatic cysts have been reported [5]. Spinal cysts are classified as extradural or intradural cysts [4]. Extradural cysts (of non-meningeal origin) are recognized in three types: (1) synovial cysts containing a lining of synovial-like epithelial cells, (2) ganglion cysts, originating from vertebral ligaments, consisting of a collagenous capsule surrounding myxoid material and (3) discoid cysts originating from the *annulus fibrosus* containing degenerative fibrous material without a synovial lining [3, 4]. Intradural cysts arise from the meninges and contain cerebrospinal fluid (CSF). These include arachnoid cysts that lack epithelial lining. For this reason they are not true cysts and the term spinal arachnoid diverticula (SAD) is more appropriate [1, 2, 6]. Meningeal cysts/diverticula can also be presented with an extradural position.

Lowrie et al. [1] introduced a classification scheme used for humans into the veterinary literature. Based on pathology, meningeal cysts were classified as type I: extradural cyst without spinal nerve root involvement, whereof type Ia is thought to arise from herniation of the arachnoid through a dural defect and type Ib is defined as meningocele connected by a pedicle to the caudal tip of the dural sac forming a dural diverticula. Type II extradural arachnoid diverticula with nerve root involvement are also known as Tarlov cysts and are presented as CSF filled dilatations between the perineum and endoneurium. Type III are intradural arachnoid diverticula without epithelial cell lining and they communicate freely with the subarachnoid space [1, 4, 7].

Spinal cysts and diverticula in humans have been reported to occur primarily in the lumbar spine [8, 9], whereas in veterinary literature both non-meningeal cysts, meningeal type II cysts, and SAD were reported to occur at two main locations; the cervical spine and thoracolumbar junction [1–4, 10, 11]. Synovial cysts have also been reported in the lumbosacral junction [4, 12, 13]. In French Bulldogs, SAD have only been reported in the thoracolumbar region [10]. To our knowledge, types Ia and Ib have not been reported in dogs.

In this case report we describe two French Bulldogs, one presenting with a meningeal cyst type Ib and one with a type III (intradural arachnoid diverticula) in the caudal lumbar area. The clinical and MRI findings, surgical treatment, histopathology, and follow up are reported.

## Case presentation

### Case 1

**Fig. 1:**
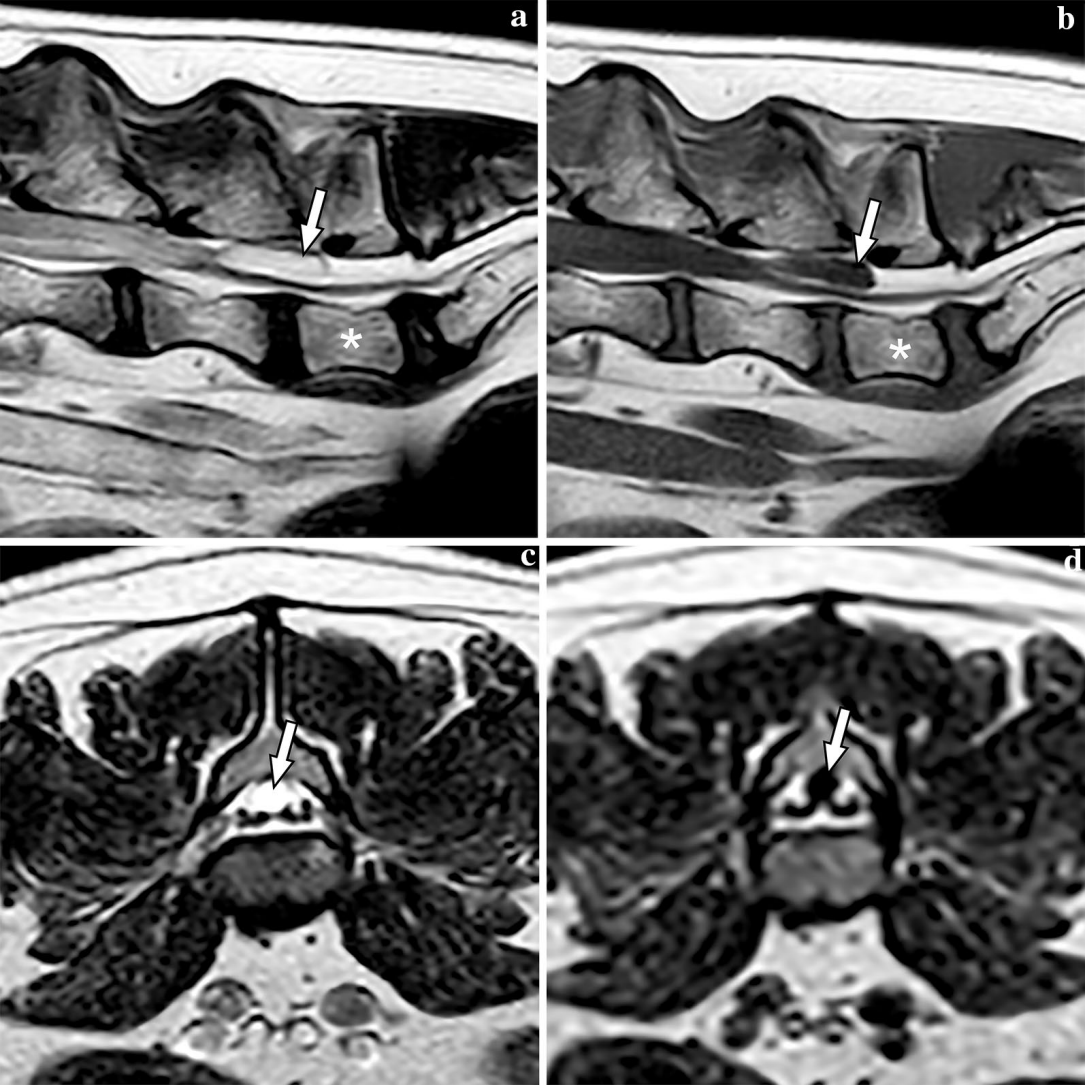
Magnetic resonance images of the caudal lumbar vertebral column of Case 1, including T2-weighted sagittal (**a**), T1-weighted sagittal (**b**), T2-weighted transverse (**c**), and FLAIR transverse (**d**) images. The meningeal cyst is marked with an arrow on all images and L7 is labelled with an asterisk on images (**a**) and (**b**)

An 8-year-old female French Bulldog weighing 12.4 kg was referred to the Department of Clinical Sciences of Companion Animals at Utrecht University with a history of intermittent faecal incontinence and progressive abnormal gait in the hind limbs since 3 months. Conservative treatment with corticosteroids initially showed improvement but after tapering the dosage, clinical signs recurred. The owners felt that the dog was in pain because they found the dog progressively more depressed/lethargic. On general examination, the dog showed signs related to brachycephalic obstructive airway syndrome. Orthopaedic examination elicited pain on palpation and extension of the L6 to S3 region. Neurological examination identified hind limb ataxia and ambulatory paraparesis and postural deficits in the hind limbs, more severe on the left side. Spinal reflexes were normal in all limbs. The neuroanatomic localization based on the neurological deficits included T3–L3 and based on the lumbosacral pain on palpation and faecal incontinence it was extended until S3. Magnetic resonance imaging (MRI, type Ingenia 1.5T, Philips, Eindhoven, The Netherlands) was performed under general anaesthesia of the thoracolumbar, lumbar and lumbosacral spine extending from T6 to Cd3 with sagittal and/or transverse T1-weigted, T2-weighted, T2*-weighted, fluid attenuation inversion recovery (FLAIR) and short tau inversion recovery (STIR) sequences (Fig. 1). At the level of L6–L7 an oval cyst-like structure was seen with dorsal compression of the cauda equina. The content of this structure was isointense with CSF. An ill-defined T2W hyperintensity was present within the spinal cord at the level of T12, most likely consistent with oedema and less likely fibrocartilaginous embolism due to the insidious onset and progressive ataxia. Kyphosis and multiple thoracic hemivertebrae were noted, consistent with breed related vertebral column deformities, without evidence of spinal cord compression. The radiological diagnosis was a cyst-like structure with dorsal compression of the cauda equina. In addition, all visible intervertebral discs showed decreased hypointense signal compatible with intervertebral disc degeneration. Mild disc protrusion was found at multiple levels, but there was no disc-associated compression of the spinal cord or cauda equina.

**Fig. 2:**
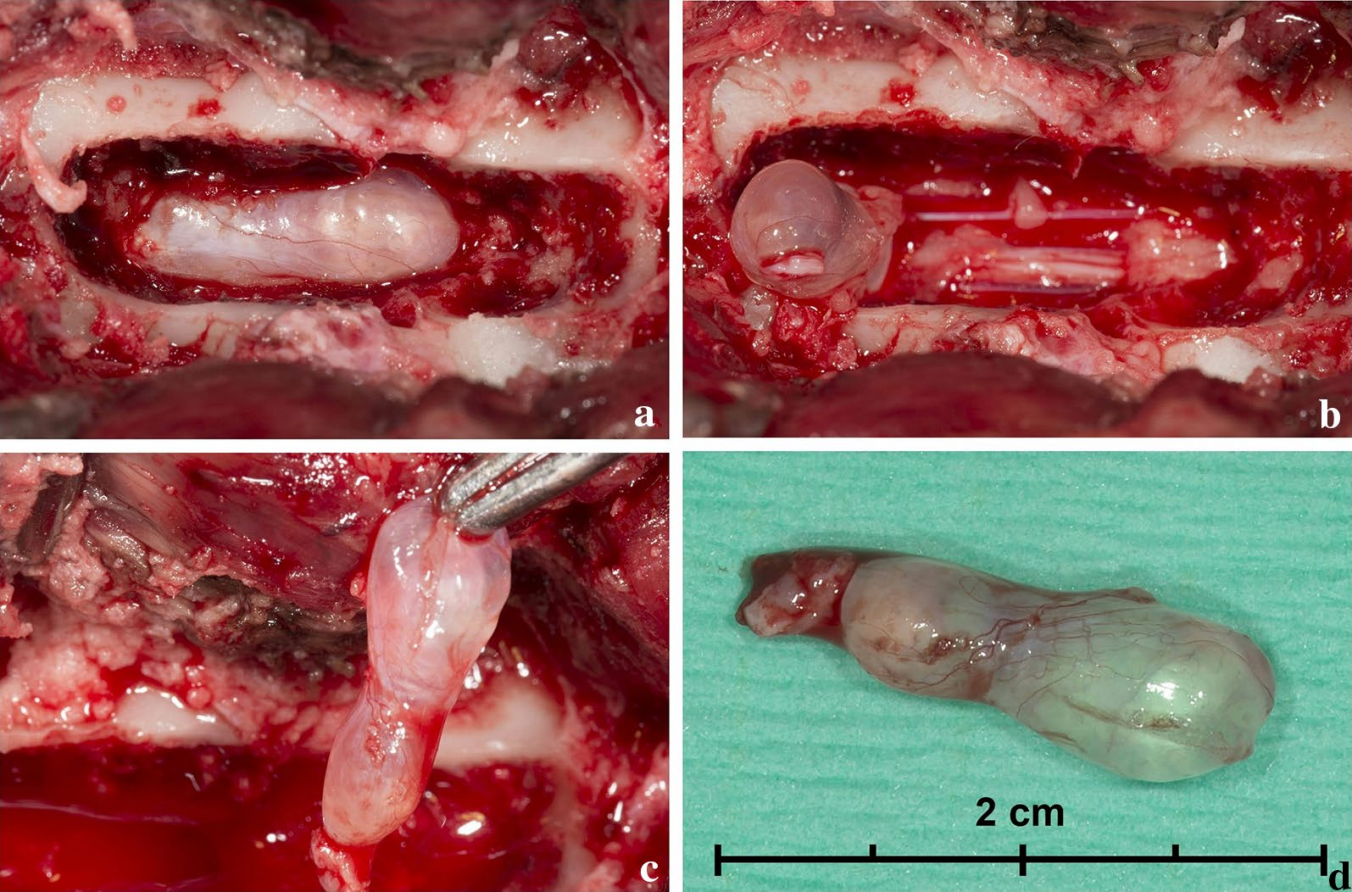
Intraoperative photographs of the surgical removal of the meningeal cyst in Case 1. Dorsal laminectomy at L6–L7 revealed a spinal cyst (**a**). The cyst was released from the cauda equina caudally (**b**) and retracted cranially (**c**) before being removed completely without collapse (**d**)

Since the dog was not responsive to medication, the owners opted for surgery. The dog received premedication with dexmedetomidine (4 µg/kg, IV) and butorphanol (0.1 mg/kg, IV). Propofol (2 mg/kg, IV) was used to induce anaesthesia. General anaesthesia was maintained with isoflurane, ketamine (10 µg/kg/min, IV) and dexmedetomidine (1 µg/kg/h, IV). Medication included cefazolin (20 mg/kg, IV), buprenorphine (10 µg/kg, IV), and dexamethasone (0.2 mg/kg, IV). A standard dorsal laminectomy from the midpoint of L6 extending over L7 to S1 was performed as described previously [14], using a high speed burr and Kerrison rongeurs. After removal of the *ligamentum flavum* with a beaver scalpel knife, the cystic structure was exposed (Fig. 2a). The cyst was carefully freed with a ball-tipped probe, the cranial side of the cyst was attached to the dural sac and the caudal side of the cyst was attached to one of the smaller caudal nerves of the cauda equina (Fig. 2b). The appearance of this caudal nerve was similar to the other nerves. The cyst was sharply dissected from the caudal nerve and the dural sac (Fig. 2c). The lumen of the cyst was not continuous with the dural sac and therefore the cyst could be removed completely without collapse (Fig. 2d). Before routine closure, a morphine splash-block was deposited in the surgical field and an autologous free fat graft was transplanted over the neural tissue to prevent dural adhesions [15].

**Fig. 3:**
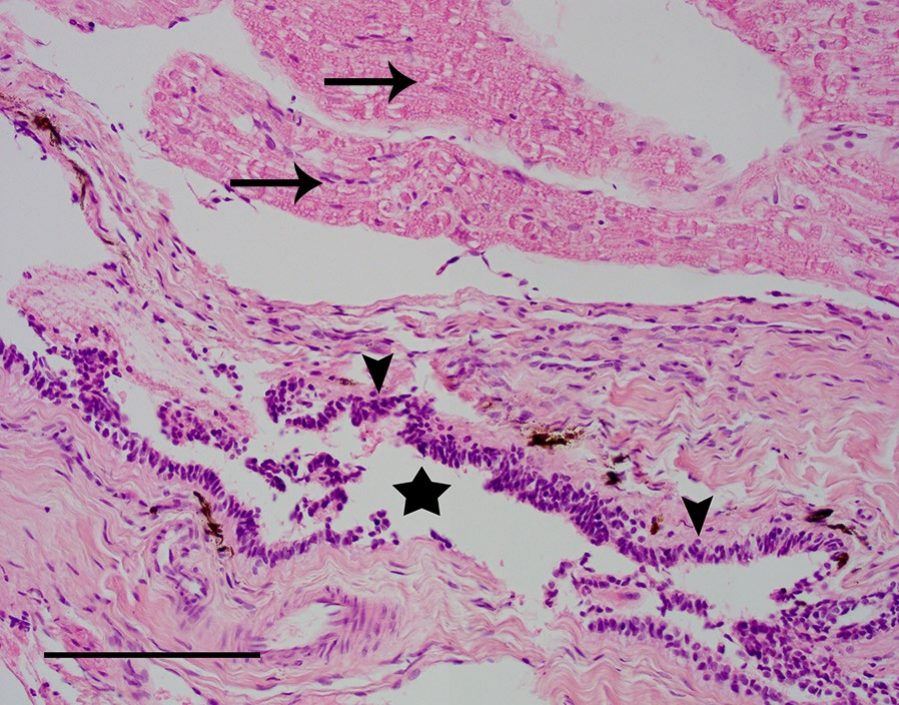
Histopathology images of Case 1. Collapsed optic empty cyst (asterisk) lined by cuboidal to cylindrical epithelium (arrowheads), surrounded by fibrous tissue. Peripheral nerve tissue (arrow) is shown at the top of the image. HE staining, bar = 100 µm

The entire cyst was fixed in 10% neutral buffered formalin, processed by routine methods *in toto*, embedded in paraffin, sliced and stained with haematoxylin and eosin (HE) and periodic acid Schiff (PAS). Macroscopically, the cyst was stalked, thin-walled, measured 1.3 × 0.5 cm and filled with clear fluid. Histologically, several optically empty slit-like spaces were lined with cuboidal epithelium and embedded in connective tissue adjacent to a peripheral nerve that showed minor degenerative changes (Fig. 3). There was sporadic perineural and perivascular lymphoplasmacellular infiltrations. After PAS staining, cilia were not detected in the epithelial lining. Thus, the histological findings were compatible with a meningeal cyst type Ib of the classification scheme proposed by Lowrie et al. [1].

The dog was discharged 1 day after surgery with the following oral medication: tramadol (2–5 mg/kg, q6–8 h), gabapentin (10 mg/kg, q12 h) and prednisolone was tapered down and stopped over a period of a few weeks. The owners were advised to keep the dog quiet and on leash walks for at least 6 weeks. Physical therapy was started at 2 weeks after surgery to support rehabilitation.

**Fig. 4:**
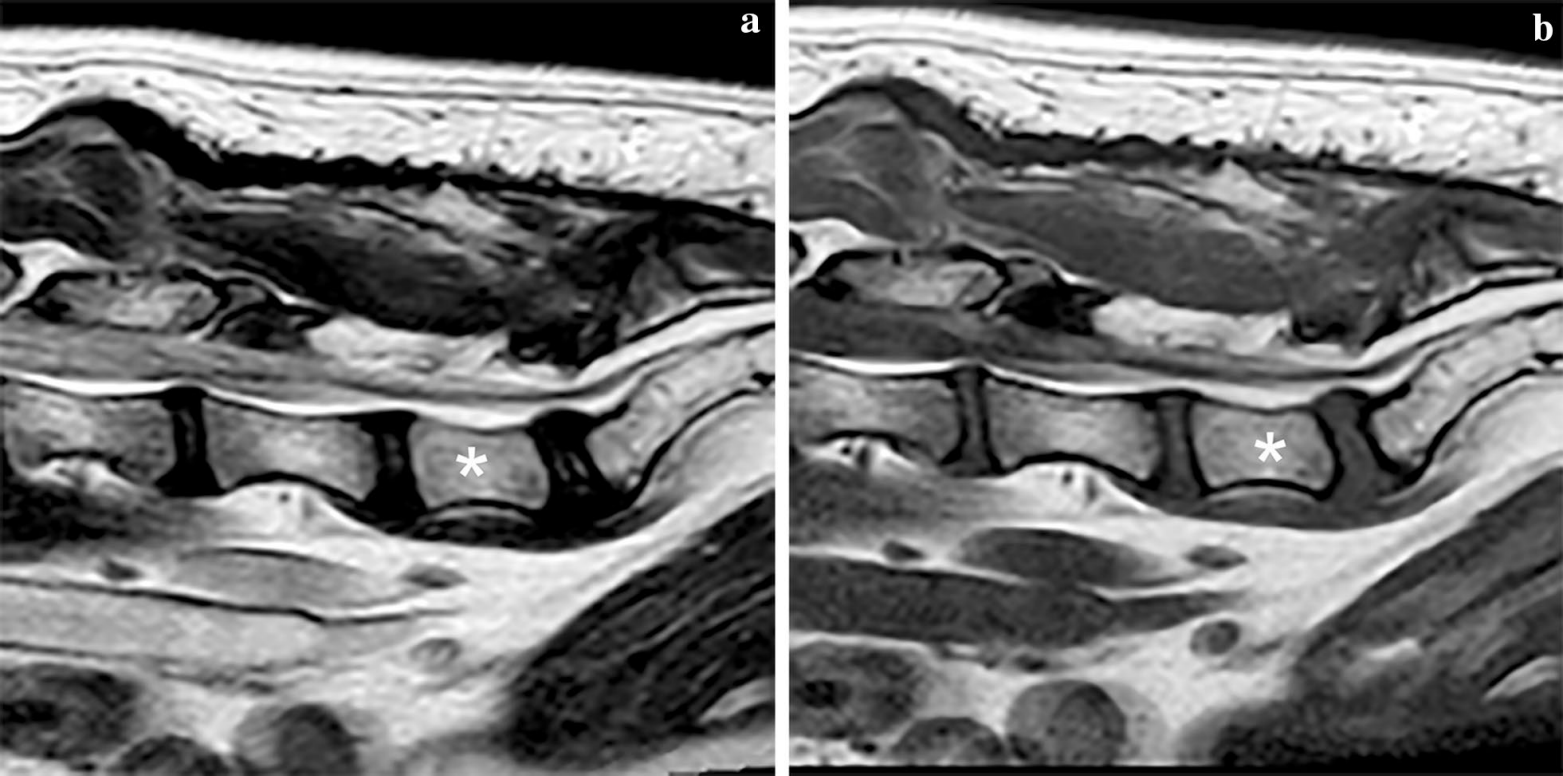
Magnetic resonance images of the caudal lumbar vertebral column of Case 1 post surgery, including T2-weighted sagittal (**a**) and T1-weighted sagittal (**b**) images. L7 is labelled with an asterisk. The meningeal cyst is absent and post-surgical changes are seen in the soft tissues dorsal to the region of interest. N.B. The oval structure at approximately the previous position of the meningeal cyst (see Fig. 1a, b) is a fat graft (T1-weighted and T2-weighted hyperintense)

At 3 months follow-up examination, the dog was without medication and the owners had noted progressive improvement in the clinical signs. The hind limb ataxia was still noticeable, but less severe and the same was true for the faecal incontinence. The dog no longer showed signs of depression/lethargy, ambulatory paraparesis was no longer present, and no pain could be elicited during palpation and/or extension of the lower back. MRI of the thoracic, lumbar and lumbosacral spine was performed, including sagittal and transverse T1W and T2W sequences, transverse T2 * W and sagittal STIR sequences (Fig. 4). The rounded cystic structure, as described in the preoperative MRI examination, was no longer visible. Dorsal to the cauda equina there was hyperintensity visible at the level of L6–L7 and the LS-junction over a length of 19.6 mm (at both T1W and T2W sequences) consistent with the fat graft which slight displaced the cauda equina ventrally [15]. The soft tissue on the dorsal side of the L6–L7–S1 spine showed irregularity and the muscles were heterogeneously hyperintense, consistent with changes secondary to surgery (Fig. 4).

### Case 2

A 5-year-old male French Bulldog weighing 10.3 kg was referred to the Department of Clinical Sciences of Companion Animals at Utrecht University with a 3-year history of hind limb ataxia and ambulatory paraparesis without clinical signs of pain. MRI of the cervical and thoracolumbar spine performed 1 year previously in another institution showed intervertebral disc degeneration at multiple levels and mild disc protrusion at T12–T13 but without compression of neural structures. The dog was treated conservatively with corticosteroids without clinical improvement. Ambulatory paraparesis persisted and in addition to the motor deficits, passive urine incontinence developed not related to overflow. Results of initial physical examination were unremarkable; however, a complete neurological examination revealed ataxia with dysmetria and hypermetria of both hind limbs, as well as muscle atrophy and hypotony. The dog had proprioceptive deficits in both hind limbs, but spinal reflexes were normal in all limbs. Based on the neurological deficits, the urinary incontinence, and the results of neurological examination, the neuroanatomic localization was determined as T3–S3.

**Fig. 5:**
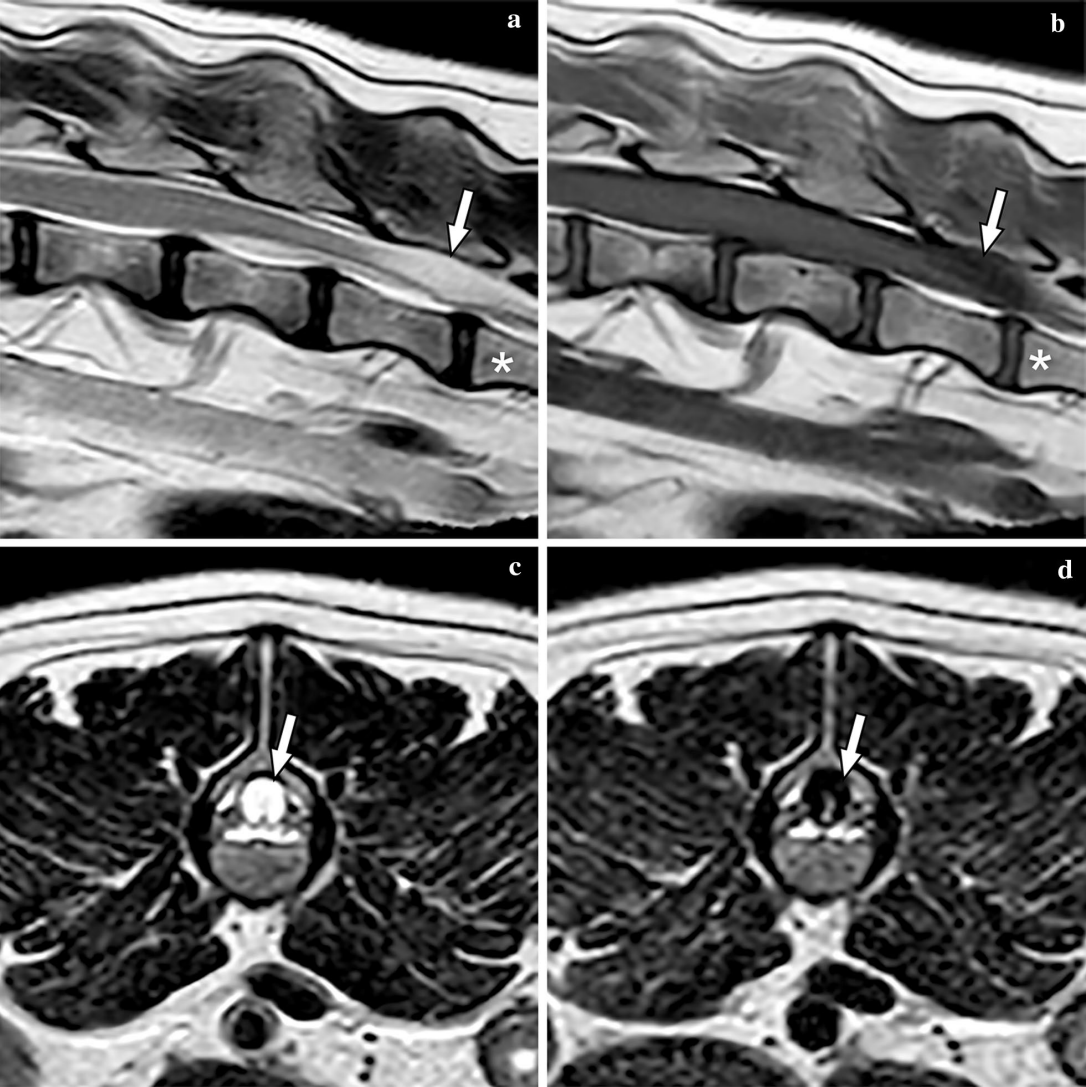
Magnetic resonance images of the caudal lumbar vertebral column of Case 2, including T2-weighted sagittal (**a**), T1-weighted sagittal (**b**), T2-weighted transverse (**c**), and FLAIR transverse (**d**) images. The subarachnoid diverticula is marked with an arrow on all images and L7 is labelled with an asterisk on images (**a**) and (**b**)

Magnetic resonance imaging was repeated from C1 to S1, including sagittal and transverse T1-weigted and T2-weighted sequences, and a transverse FLAIR sequence of the lumbar spine. Multiple intervertebral discs showed decreased hypointense signal compatible with intervertebral disc degeneration. At the level of C2–C3, T12–T13, T13–L1 and L1–L2 mild disc protrusion was found with visible compression of the ventral subarachnoid space, but without compression or dislocation of the spinal cord. A fusiform cystic lesion dorsal to the cauda equina at the level of L6–L7 was found with an ill-defined cranial and caudal border. The cystic structure was isointense to CSF and compressed the cauda equina (Fig. 5). Based on these imaging characteristics a SAD was suspected. There was mild dilation of the central canal from T12 to L5.

Anaesthesia and medication for the surgical procedure were identical to Case 1 except that this dog received carprofen (4 mg/kg, IV) instead of corticosteroids in the postoperative period. The surgical technique was the same as for Case 1. After dorsal laminectomy from halfway L6 over L7 to S1, a dilated dural sac was exposed which was macroscopically identified as SAD. A durotomy was performed over a length of approximately 3 cm, which resulted in free flow of CSF into the spinal canal and collapse of the diverticula. No tissue was available for histological examination. Marsupialization of the SAD was performed by placing interrupted sutures with polydioxanone 5-0 attaching the dural sac to the lateral wall of the laminectomy. Before closure the laminectomy was covered with an autologous fat graft to prevent adhesions.

The dog was discharged 1 day after surgery with the following oral medication: amoxicillin/clavulanic acid (12.5 mg/kg, q12 h), tramadol (2–5 mg/kg, q6–8 h), and carprofen (2 mg/kg q12 h). The owners were advised to keep the dog quiet and on leash walks for 6 weeks.

The dog was re-examined at 2 weeks after surgery. The dog showed moderate ataxia and postural reaction deficits in the hind limbs. The dog was both urinary and faecal continent. All spinal reflexes including perineal reflex were normal. Physical therapy and hydrotherapy was started to support rehabilitation. At 6 weeks postoperatively, the motor function of the dog had recovered significantly. Although, the dog was still ataxic, the dog was evidently less paretic in the hind limbs. Postural deficits were improved in both hind limbs and all spinal reflexes were normal. Also, the muscle mass over the hind limbs had progressively increased.

## Discussion and conclusions

In this study, a meningeal cyst and a SAD are reported in the lumbar region of French Bulldogs. Although spinal cysts have been described more frequently in veterinary literature in recent years, prevalence of canine arachnoid diverticula in the caudal lumbar region is very rare [1, 10]. Schmöckel and Rapp [13] described a case series of synovial cyst at the lumbosacral junction in three German Shepherd dogs. One case, reported by Webb et al. [12] and two dogs of a case series reported by Sale and Smith [16], presented a synovial/ganglion cyst associated with the L6–L7 articulations. Here we describe a case of a meningeal spinal cyst type Ib and a case of a type III spinal arachnoid diverticula at the L6–L7 region, which both have not been reported before. Although the aetiology of spinal cysts still remains unclear, theories are different between the various type of cysts/diverticula. Extradural cysts (of non-meningeal origin) were linked to increased mechanical stress and instability [2, 4, 12, 13, 16], whereas meningeal cysts are generally considered congenital lesions with a genetic predisposition [1–4, 10, 11]. However, SAD may also be acquired or traumatic, and seems to be related to concurrent spinal disorders like vertebral malformations and intervertebral disc disease [1, 10].

Congenital vertebral column deformities like hemivertebrae and degenerative intervertebral disc disease have been reported previously in French Bulldogs and other chondrodystrophic breeds [17, 18]. In agreement with previous studies, instability is suspected to play an important role as possible underlying cause of the evolution of lumbar spinal cyst [3, 4, 10]. In the French Bulldogs in our case series, congenital vertebral abnormalities, i.e. kyphosis and hemivertebrae and intervertebral disc disease, were also evident on MRI and it is likely that the aetiology and evolution of the lumbar spinal cysts is in some way genetically linked to spinal deformity and abnormal biomechanics of the spine in French Bulldogs. Mauler et al. [10] found high rates (61.5%) of concurrent diseases in French Bulldogs with SAD [10] and a predisposition to acquired SAD was presumed [1, 4, 10] resembling our Case 2.

Progressive postoperative clinical improvement suggested the causality between the cyst and some of the clinical signs, although this is difficult to confirm due to the absence of a control group. Both cases showed clinical signs consistent with a neuroanatomical localization T3–L3 (hind limb ataxia and paraparesis, postural deficits of the hind limbs, and normal spinal reflexes represent upper motor neuron signs related to spinal cord segments), and could be further extended until S3 (pain reaction on palpation and extension of the L6 to S3 segment, paraparesis, intermittent faecal incontinence in Case 1, and passive urinary incontinence in Case 2, represent lower motor neuron signs related to cauda equina). Persistent neurological deficits, i.e. moderate hind limb ataxia and, although less frequent, persistent intermittent faecal incontinence (Case 1) may be explained by two possible reasons. First, clinical signs of hind limb ataxia may be explained by other detected spinal deformities, i.e. the intramedullary hyperintensity at the level of T12 representing spinal cord oedema, and abnormalities common to the brachycephalic breed, i.e. hemivertebrae, kyphosis, and degenerative disc disease [19]. In a recent publication, Ryan et al. [20] studied in retrospect neurologically normal French Bulldogs and found a high prevalence of vertebral malformations. In our cases, we could not exclude any clinical relation to this finding (our patients both showed neurological deficits); however, since vertebral column deformities in French Bulldogs are common incidental findings, it appears to be less likely. In the first case, the MRI study extended from T6 to Cd3, abnormalities cranial to the T6 segment which could be related to the neurological signs like ataxia could not be excluded. In the second case, the MRI study extended from C1 to S1. Multiple mild protrusions of intervertebral discs at the cervical, thoracic and the lumbar regions were seen, but without evident compression of the spinal cord. Therefore, we cannot completely exclude a possible relation of some of the neurological signs to the intervertebral disc protrusions, but it seems less likely since there was no significant compression of the spinal cord or cauda equina. Also, the occurrence of a spinal cyst is usually a gradual and chronic problem; therefore the cyst may have affected the dural sac and the cauda equina nerves already for a prolonged time, leading to permanent changes in the neural tissue. It is not possible to state whether the spinal cyst/diverticula, the concurrent spinal disorder, or a combination of both attributed to the neurologic signs. Although, it remains unexplained why the dogs did not recover completely, both dogs showed an acceptable clinical outcome and the owners were satisfied with the result.

In accordance to radiological, intra-operative and histopathological findings our first case was categorized as an extradural meningeal type Ib cyst [1, 4]. Type Ib spinal cysts are closely related to myelomeningocele for which Bulldogs are predisposed [1]. Only few case reports [17, 18] have reported myelomeningocele. Ployart et al. [17] described a case of myelomeningocele and a dermoid sinus-like lesion at the L7 site in the French Bulldogs, which is different from our clinical case. Whether the cyst in Case 1 was a type Ib or a type II (perineural or Tarlov cyst) [1] remains under discussion. Histopathological findings showed some infiltrations of perineural tissue, but there was no clear evidence of nerve root involvement. Therefore a type Ib defined as meningoceles connected by a pedicle to the caudal tip of the dural sac forming a dural diverticulum [1] is the most likely histological diagnosis. In Case 2 there was no tissue available for histology, but radiologic and intra-operative findings demonstrated a type III intradural arachnoid diverticula [1]. A type Ib spinal cyst (case 1) has not been reported previously in dogs [1] and SAD (case 2) has not been reported in the caudal lumbar (L6–L7) region in dogs [10, 21].

In conclusion, this is the first report of caudal lumbar spinal cysts/diverticula in French Bulldogs and gives us more knowledge and insights into (congenital) spinal deformities and abnormalities related to the French Bulldogs breed. Spinal cysts/diverticula should be included in the differential diagnosis of cauda equina syndrome and lower back pain in French Bulldogs. However, further studies are indicated to evaluate the prevalence and possible genetic relation of spinal cysts/diverticula to the French Bulldog breed.

## Abbreviations

CSF: cerebrospinal fluid
CT: computed tomography
FLAIR: fluid attenuation inversion recovery
HE: haematoxylin and eosin
IV: intravenous
MRI: magnetic resonance imaging
PAS: periodic acid Schiff
SAD: spinal arachnoid diverticula
STIR: short tau inversion recovery
